# Molecular evolution of the human *SRPX2 *gene that causes brain disorders of the Rolandic and Sylvian speech areas

**DOI:** 10.1186/1471-2156-8-72

**Published:** 2007-10-18

**Authors:** Barbara Royer, Dinesh C Soares, Paul N Barlow, Ronald E Bontrop, Patrice Roll, Andrée Robaglia-Schlupp, Antoine Blancher, Anthony Levasseur, Pierre Cau, Pierre Pontarotti, Pierre Szepetowski

**Affiliations:** 1 INSERM UMR 491, Université de la Méditerranée, 13385 Marseille, Cedex 5, France; 2 Edinburgh Biomolecular NMR Unit, The University of Edinburgh, Edinburgh EH9 3JJ, UK; 3 Biomedical Primate Research Centre, 2280 GH Rijswijk, The Netherlands; 4 LIGM, Université Paul Sabatier, 31403 Toulouse, Cedex 4, France; 5 EA 3781, Université d'Aix-Marseille I, 13331 Marseille, Cedex 3, France

## Abstract

**Background:**

The X-linked *SRPX2 *gene encodes a Sushi Repeat-containing Protein of unknown function and is mutated in two disorders of the Rolandic/Sylvian speech areas. Since it is linked to defects in the functioning and the development of brain areas for speech production, *SRPX2 *may thus have participated in the adaptive organization of such brain regions. To address this issue, we have examined the recent molecular evolution of the *SRPX2 *gene.

**Results:**

The complete coding region was sequenced in 24 human X chromosomes from worldwide populations and in six representative nonhuman primate species. One single, fixed amino acid change (R75K) has been specifically incorporated in human SRPX2 since the human-chimpanzee split. The R75K substitution occurred in the first sushi domain of SRPX2, only three amino acid residues away from a previously reported disease-causing mutation (Y72S). Three-dimensional structural modeling of the first sushi domain revealed that Y72 and K75 are both situated in the hypervariable loop that is usually implicated in protein-protein interactions. The side-chain of residue 75 is exposed, and is located within an unusual and SRPX-specific protruding extension to the hypervariable loop. The analysis of non-synonymous/synonymous substitution rate (Ka/Ks) ratio in primates was performed in order to test for positive selection during recent evolution. Using the branch models, the Ka/Ks ratio for the human branch was significantly different (p = 0.027) from that of the other branches. In contrast, the branch-site tests did not reach significance. Genetic analysis was also performed by sequencing 9,908 kilobases (kb) of intronic *SRPX2 *sequences. Despite low nucleotide diversity, neither the HKA (Hudson-Kreitman-Aguadé) test nor the Tajima's D test reached significance.

**Conclusion:**

The R75K human-specific variation occurred in an important functional loop of the first sushi domain of SRPX2, indicating that this evolutionary mutation may have functional importance; however, positive selection for R75K could not be demonstrated. Nevertheless, our data contribute to the first understanding of molecular evolution of the human *SPRX2 *gene. Further experiments are now required in order to evaluate the possible consequences of R75K on SRPX2 interactions and functioning.

## Background

Evolution studies have been undertaken to identify those genetic changes that underlie human-specific features such as susceptibility to acquired immunodeficiency syndrome, bipedalism, a large brain, and higher-order cognitive functions. Several phenotypic differences distinguishing human from other great apes species obviously rely on cerebral activity. Large-scale studies in human and chimpanzee using either genome comparisons [1,2] or brain transcriptome analyses [3-5] have led to the identification of a subset of genes that may have contributed to the evolution of human brain anatomy and activity from a common primate ancestor. An important complementary approach has relied on the study of candidate genes selected on the basis of their importance in specific human phenotypes. Consequently, several genes involved in the structure and/or functioning of the human brain have been associated with recent positive selection: *ASPM *[6,7], *MCPH1 *[8-10], *GLUD2 *[11], *MAOA *[12,13], *SHH *[14], and the "speech gene" *FOXP2 *[15-17]. More recently, accelerated evolution of noncoding sequences has also been shown [18,19].

The Rolandic and Sylvian fissures divide the cortex hemispheres of primates into their main anatomical structures. In human, these areas participate in speech production under the control of the Broca's area. We recently identified the *SRPX2 *gene as being responsible for two related disorders of the Rolandic and Sylvian speech areas [20,21]. Since it is linked to defects in the functioning and the development of such brain regions, such as epileptic seizures, oral and speech dyspraxia, or bilateral perisylvian polymicrogyria, *SRPX2 *may be one of the specific genes whose evolution at the DNA-level may have participated in the recent emergence of higher-order cognitive functions, including the adaptive organization of brain areas for speech production.

In this study, we have examined the molecular evolution of the *SRPX2 *gene. One single, fixed amino acid change occurred in the first sushi domain (also known as CCP – complement control protein – module, or short consensus repeat) of SRPX2 after the human-chimpanzee split. Three-dimensional modeling showed that both this evolutionary mutation and a previously identified disease-associated mutation [20] lie within a hypervariable loop shared by all sushi modules and that has been implicated in some cases in protein-protein interactions [22]. Using the branch models, the synonymous/non-synonymous analysis was consistent with accelerated evolution in the human lineage but this could not be confirmed when the branch-site models were used. Population genetics tests did not reach statistical significance, indicating either that a selective sweep may have occurred more than 100 000–200 000 years ago, or that there has been no episode of positive selection on *SRPX2*.

## Results and discussion

### One single amino acid substitution (R75K) has been fixed in human SRPX2 since the human-chimpanzee split

**Figure 1:**
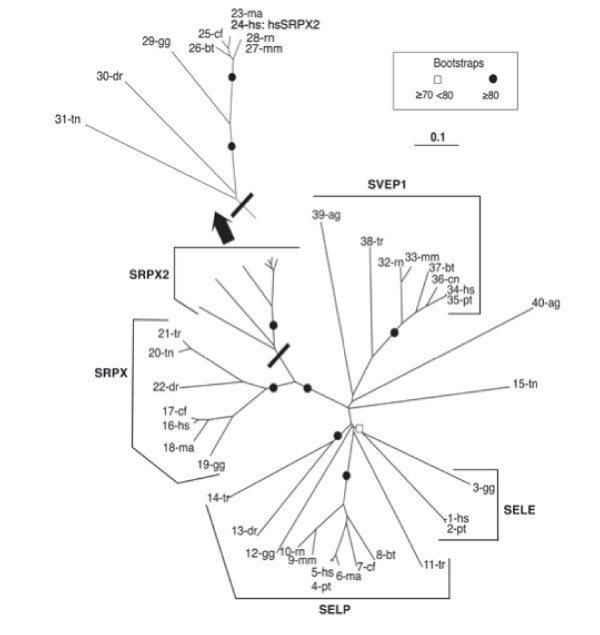
Phylogenetic tree of the SRPX2 family members. ag: Anopheles gambiae; bt: Bos taurus; cn: Canis familiaris; dr: Danio rerio; gg: Gallus gallus; hs: Homo sapiens; mm: Mus musculus; ma: Macaca mulatta; rn: Rattus norvegicus; pt: Pan troglodytes; tn: Takifugu rubripes; tr: Tetraodon nigroviridis. The list of the ENSEMBL gene accession numbers used to construct the phylogenetic tree is available in Additional file 1.

**Figure 2:**
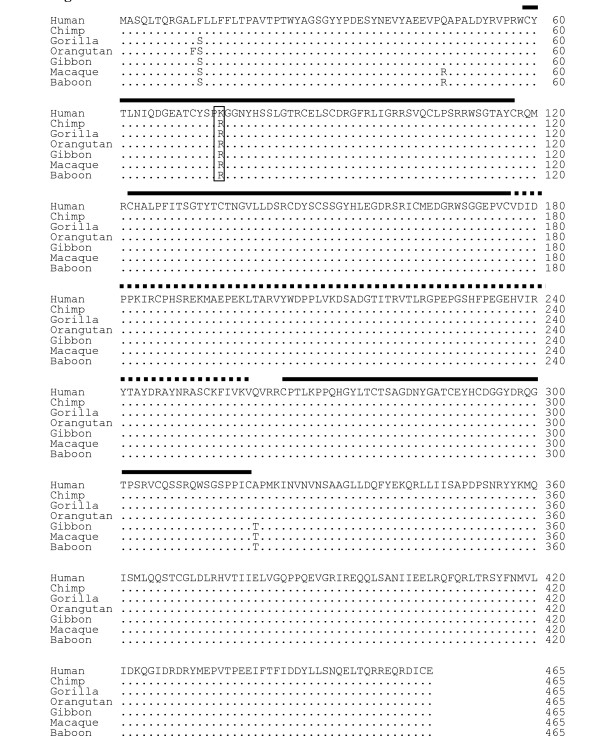
Amino acid sequence alignment of SRPX2 from human and nonhuman primates. Dots represent residues identical to the human amino acid sequence. Black lines represent the three Sushi domains and the dotted line represents the HYR domain. The site of the human-specific variation (R75K) is boxed.

*In silico *screening of publicly available databases followed by phylogenetic analysis showed that *SRPX2 *belongs to a family of five genes: *SRPX2 *itself (sushi-repeat-containing protein, X-linked 2), *SRPX *(sushi-repeat-containing protein, X-linked), *SELP *(selectin P precursor), *SELE *(selectin E precursor), and *SVEP1 *(selectin-like protein) gene (Fig. 1; Additional file 1). This family emerged during vertebrate evolution. In order to trace the recent evolutionary history of *SRPX2*, all coding exons were sequenced from a subset of primate species that represent key evolutionary steps: human [Genbank: NM_014467], chimpanzee [Genbank: EF369515], gorilla [Genbank: EF369516], orangutan [Genbank: EF369517], gibbon [Genbank: EF369518], macaque [Genbank: EF369519], and baboon [Genbank: EF369520]. Sequence comparison (Fig. 2) detected only five amino acid variant sites (1.1%; 5/465). One discrepancy between the protein [Genbank: ABN46998] predicted from the five chimpanzee SRPX2 sequences obtained here (see Methods section), and the chimpanzee SRPX2 protein [Genbank: XP_521170] predicted from the genomic sequence at the UCSC database [23], was detected (D429 in ABN46998; N429 in XP_521170). This likely corresponds to an error in the chimpanzee genome sequence previously available, although it may be due to chimpanzee polymorphism. The only amino acid substitution specific to the human lineage is an Arg to Lys change at position 75, corresponding to a 224A>G mutation within exon 4 of *SRPX2*. The possible importance of the R75K substitution in the evolution of the human species was indicated by the conservation of R75 in all SRPX2 orthologues, from nonhuman primates to ray-finned fishes, as well as by the fixation of K75 in human, as demonstrated by the lack of any variation at position 75 *in silico*, in the 24 worldwide X-chromosomes tested here, and in the 624 Caucasian control X-chromosomes previously screened [20].

### K75 is situated in the hypervariable loop that is usually implicated in protein-protein interactions

The R75K human-specific modification occurred in the first sushi domain of the protein. Sushi domains have been identified in several proteins of the complement system and in the selectin family of proteins [24]. They may serve in protein-protein interactions [22], as demonstrated in the case of the neurocan-L1 interaction [25]. R75K occurs only three amino acid residues away from the tyrosine residue (Y72) that is mutated in a patient with rolandic seizures and bilateral perisylvian polymicrogyria and in his female relatives with mild mental retardation [20]. The amino acid residue at position 75 may thus participate in the proper function of the first sushi domain of SRPX2.

**Figure 3:**
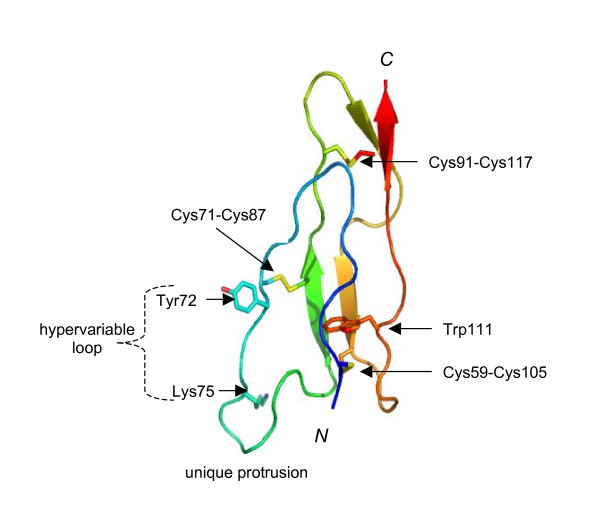
Three-dimensional modeling of the first sushi domain of human SRPX2. A cartoon representation [65] is shown, highlighting the conserved Trp and Cys residues. The model reveals an additional putative disulfide bridge (Cys71-Cys87), atypical of the classical sushi (CCP) module fold. The hypervariable loop, with the sites of disease-causing mutation (Tyr72) and evolutionary change (Lys75) and the protrusion specific to SRPX, are indicated.

To further address this issue, three-dimensional modeling of the first sushi module of the human SRPX2 protein (*i.e*. sushi 1) was undertaken by homology with a known sushi domain (CCP module) structure (Fig. 3; Additional file 2). Sushi domains are characterised by a compact hydrophobic core, containing an almost invariant Trp residue, which is enclosed in a framework of five extended segments that form β-strands for all or part of their lengths. The extended segments are aligned with the long axis of the module and are held together by two strictly conserved disulfide bridges [24]. In a previously performed cluster assignment exercise undertaken for more than 240 sushi domain sequences [26], the first sushi domain of SRPX (which is highly similar to sushi 1) fell into cluster-D, which also includes many modules found within the selectin family of proteins.

In almost all sushi domains, a region that is highly variable in length, sequence and conformation, and commonly referred to as the hypervariable loop [27], is inserted within the second extended region. Depending on its length this projects laterally from the module and forms an obvious candidate surface for protein-protein recognition. Indeed, the hypervariable loop has been implicated as a "hot-spot" for several protein-protein interactions and disease-causing mutations in CCP-containing complement proteins [22,28-30]. In the sushi 1 model (Fig. 3) an additional disulfide bridge (cysteine residues C71-C87), atypical of the classical sushi domain fold, is evident. Cys71 lies at the beginning of the hypervariable loop that is exceptionally long in sushi 1. The hypervariable loop is thus forced to form a prominent protrusion extending towards the N-terminus of the module. This feature has neither been seen in any experimentally determined sushi module structure to date, nor is predicted to occur in other members of the D-cluster except SRPX.

The Y72S mutation is largely solvent exposed and located within the hypervariable loop, adjacent to the cysteine residue (C71) that participates in the non-typical, third, disulfide bridge. This change from a large aromatic side-chain (tyrosine; Y) to a small, polar one (serine; S) at position 72 will have a profound effect on the surface properties of this region that is close to the aforementioned prominent protrusion. K75 is located nearby, within the protrusion, and its side-chain is exposed (Fig. 3). It is reasonable to suggest that the unique structure formed by the hypervariable loop of sushi 1 performs a role that is specific to the SRPX2 protein. Presumably, it is not a coincidence, that it is also the site of the human-specific change. An R75K substitution is a conservative one; the substitution of one exposed, positively charged residue for another can easily be accommodated by small atom shifts in surrounding side-chains, and is not likely to affect the structure of the sushi domain. Such a change might have a small but not a dramatic functional effect. Conservative substitutions are thought to play a role in adaptive change [31]. Moreover, conservative R-to-K and K-to-R substitutions can result in the altered properties of either secreted proteins [32] or the extracellular domains of some plasma membrane proteins [33,34], including a member of the selectin family [35].

### Analysis of non-synonymous/synonymous substitution rate ratio in primates

**Figure 4:**
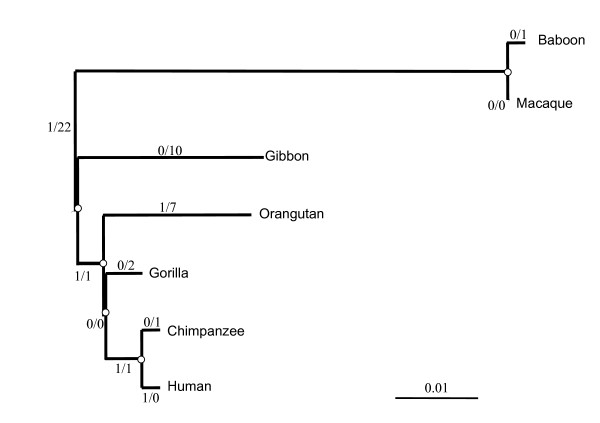
Numbers of non-synonymous (left) and synonymous (right) substitutions of different evolutionary lineages in primates.

Human-specific modifications with putative functional consequences may sustain positive selection since the human-chimpanzee split. The ratio of non-synonymous (Ka) to synonymous (Ks) substitution rates was then calculated for each branch on the primate phylogenetic tree (Fig. 4). Using the branch models, the Ka/Ks value in the human lineage differed from all other branches with statistical significance (p = 0.027) (Additional file 3). In this model, the calculated Ka/Ks ratio has an infinite value, as no synonymous change has accumulated between the human and the chimpanzee. However, this elevated Ka/Ks ratio was not statistically >1 (p = 0.371) (Additional file 3). Using the branch-site tests, model A indicated that the K75 site might be under positive selection in the human lineage. However, model A did not differ from the null hypothesis with statistical significance (p = 0.089) (Additional figure 3). Overall, positive selection in the human lineage could not be clearly demonstrated, as the branch-site analysis did not confirm the data obtained using the branch models. This may be due either to the actual absence of any positive selection, or to the lack of power of the tests due to the low number of genetic variations within *SRPX2*.

### Population genetics analysis

Evidence for positive selection can also be found by analyzing the intra-specific variation in DNA sequences subjected to a selective sweep. DNA sequences adjacent to an advantageous allele should display lower-than-expected levels of diversity. The levels of polymorphism and divergence were estimated by sequencing a total of 9,908 kb of intronic sequences surrounding exon 4 of *SRPX2 *in the 24 X-chromosomes mentioned above. Polymorphic sites are shown in Table 1. Nucleotide diversity at the *SRPX2 *locus (π = 0.00036) was 76% of the estimated nucleotide diversity on chromosome X (π = 0.00047) [36]. The divergence between humans and chimpanzee for *SRPX2 *(*D *= 0.0075) was nearly identical to the average divergence calculated for X-linked intronic regions (*D *= 0.0072) [37].

**Table 1:** Polymorphic sites of *SRPX2 *introns 3 and 4 in 12 women worldwide.

Site*	Intron 3															Intron 4		
		
	3478	3749	4292	4756	5115	6076	6783	7862	8433	9691	9700	10308	10446	10732	11286	2112	2202	2369
SNPs (ID)	a	b	c	d	e	f	g	h	i	j	k	l	m	n	o	p	q	r

Caucasian 1	T/T	C/C	C/C	C/C	C/C	C/C	G/G	A/A	G/G	A/A	T/T	T/T	T/T	C/C	G/G	T/T	C/C	A/A
Caucasian 2	T/T	C/C	C/C	C/T	C/C	C/C	G/G	A/A	G/G	A/A	T/T	T/A	T/T	C/C	G/G	T/T	C/C	A/A
Caucasian 3	T/T	C/C	C/C	C/C	C/C	C/C	G/G	A/C	G/G	A/G	T/C	T/T	T/T	C/T	G/G	T/T	C/C	A/A
Caucasian 4	T/T	C/C	C/C	C/C	C/C	C/C	G/G	A/A	G/G	A/A	T/T	T/T	T/T	C/C	G/G	T/T	C/C	A/A
Maghrebian 1	T/T	C/C	C/C	C/C	C/C	C/C	G/G	A/A	G/G	A/A	T/T	T/T	T/T	C/C	G/G	T/T	C/C	A/A
Maghrebian 2	T/G	C/C	C/C	C/C	C/C	C/C	G/G	A/C	G/G	A/G	T/C	T/T	T/T	C/C	G/G	T/T	C/C	A/A
Maghrebian 3	T/T	C/C	C/C	C/C	C/C	C/C	G/G	A/A	G/G	A/A	T/T	T/T	T/T	C/C	G/G	T/T	C/C	A/A
Asian 1	T/T	C/C	C/C	C/C	C/C	C/C	G/G	A/C	G/A	A/G	T/C	T/T	T/T	C/T	G/G	C/C	C/C	A/A
Asian 2	T/T	C/C	C/C	C/C	C/C	C/C	G/G	A/A	G/G	A/A	T/T	T/T	T/T	C/C	G/G	T/T	C/C	A/A
Asian 3	T/T	C/C	C/C	C/C	C/C	C/C	G/G	A/C	G/A	A/G	T/C	T/T	T/T	C/T	G/G	T/C	C/C	A/A
African 1	G/G	C/C	C/C	C/C	C/C	C/T	G/G	C/C	G/G	G/G	C/C	T/T	T/T	C/T	T/G	T/T	C/T	A/G
African 2	G/G	T/T	G/G	C/C	T/T	C/C	A/A	C/C	G/G	G/G	C/C	T/T	C/C	T/T	G/G	T/T	C/C	G/G

* Numbers refer to the positions in the sequences of human introns 3 and 4, respectively. ** For those single nucleotide polymorphisms (SNPs) that were not reported in current databases [23,66] at the time of analysis and that appeared in one single individual only, PCRs were redone and both strands resequenced in order to exclude PCR artifacts. SNPs IDs at NCBI [66] are: a: rs6616171. b: ss52048551. c: ss52048547. d: ss52085994. e: ss52048549. f: ss52084549. g: ss52084548. h: rs5966711. i: ss52048552. j: ss52048553. k: ss52048554. l: ss52048546. m: rs5921619. n: rs5920840. o: ss52084547. p: rs5921620. q: ss52048548. r: rs2022475.

The McDonald-Kreitman test [38] that measures the fraction of site under positive selection pressure by comparing the ratio of nonsynonymous to synonymous divergence and the ratio of nonsynonymous to synonymous polymorphism, was useless in the case of *SRPX2 *because of the very few number of inter- and intra-species DNA variations: no synonymous mutation and only one non-synonymous mutation has occurred since the human-chimpanzee split, and neither non-synonymous nor synonymous polymorphisms were detected when 24 human *SRPX2 *entire coding sequences from two sub-Saharan African, three Asian, three Maghrebian, and four Caucasian women were tested. Moreover, no polymorphism had been detected in the 198 unrelated patients previously screened for disease-causing mutations in the coding sequence of *SRPX2 *[20]. It is also noteworthy that while no synonymous polymorphism was found *in silico*, one non-synonymous variation was detected *in silico *[dbSNP:rs17851822]. Although the existence of a rare DNA variation cannot be ruled out, this change was detected in one single IMAGE clone only [Genbank: BC020733; IMAGE:4769946]. This is more likely to correspond to a clone artifact, as we indeed detected a large proportion of clones carrying artifactual mutations within *SRPX2 *inserts, using various *Escherichia coli *strains (unpublished data).

The HKA (Hudson-Kreitman-Aguadé) test [39] was then used in order to compare the intra- and inter-specific variations between the *SRPX2 *locus and control loci assumed to be under neutral selection, but did not yield significant results (Table 2). The Tajima's D test was also applied to the *SRPX2 *intron data. Tajima's D was clearly negative (D = -0.646) but did not reach significance (p > 0.05). As in the case of the *ASPM *gene that is involved in human brain expansion [6], the present intra-specific analyses did not show significant evidence against neutral expectations. The lack of statistical significance may indicate that no selective sweep has ever occurred in the human lineage. However, it should be mentioned that a selective sweep can be detected by intra-specific studies only for a short period (< 0.5 *N *generations, where *N *is the effective human population size, *i.e*. approximately < 100 000–200 000 years ago) after fixation of the advantageous variant [40,41].

**Table 2:** Nucleotide polymorphism and divergence at *SRPX2*

	Sequence length (nt)	π (%)	θ (%)	Divergence *D *(%)	θ/*D*	HKA probability
β-globin initiation at 11p15 [67]	6076	0.129	0.107	1.284	0.083	0.696
Noncoding region at 22q11 [68]	9901	0.088	0.139	1.353	0.103	0.605
Dystrophin intron-dys44 at Xp21 [69]	7475	0.135	0.102	0.604	0.169	0.499
*PDHA1 *introns at Xp22 [70]	3530	0.225	0.211	0.992	0.213	0.168
Noncoding region at Xq13.3 [71]	10200	0.045	0.083	0.922	0.090	0.655
*SRPX2 *introns at Xq22 (this study)	9908	0.048 (0.036)	0.062 (0.047)	0.750	0.084	

nt: nucleotide; π: nucleotide diversity per site; for X chromosome data, π is corrected by multiplication by 4/3 and the uncorrected value is in brackets. θ: Watterson's estimate of polymorphism per site; for X chromosome data, θ is corrected by multiplication by 4/3 and the uncorrected value is in brackets. *D*: number of nucleotide differences per site between human and chimpanzee sequences. HKA probability: probability from the HKA test, with comparison to the SRPX2 intron data.

## Conclusion

In this study, we have examined the molecular evolution of the *SRPX2 *gene that causes brain disorders of the speech areas. One single, fixed amino acid change (R75K) occurred in the first sushi domain of SRPX2 after the human-chimpanzee split. Neither the primate analysis nor the population genetics separately demonstrated the existence of positive selection of *SRPX2*. Whether the single human-specific R75K mutation has sustained positive selection thus remains an open question. However the 3-D location of R75K right within an important functional domain of the SRPX2 protein, in the immediate vicinity of a pathogenic mutation, indicates that this evolutionary mutation may have functional importance. Because R75K occurred in a domain implicated in protein-protein interactions, it is possible that qualitative or quantitative changes in the interaction with one or several putative SRPX2 partners have been modified. Despite the present lack of any knowledge on either the actual function of SRPX2 or the proteins it interacts with, it is obvious that the comparison of the properties of the human and chimpanzee SRPX2 orthologues will help determine in the future if and how new functions were acquired. From this viewpoint, our study represents a first important step towards the analysis of the consequences of R75K on SRPX2 functioning and protein interactions.

## Methods

### DNA samples and sequencing

All human and nonhuman primate DNAs were extracted using standard procedures and according to the appropriate ethical committees and animal' care rules, respectively. Polymerase chain reaction (PCR) fragments representing 9,908 kb from introns 3 and 4 were sequenced with specific primers in 12 human females from the major continental populations (two sub-Saharan Africans, four Europeans, three Asians, and three Maghrebians). Each exon of the full-length coding region of *SRPX2 *was amplified by PCR and sequenced in the same 12 women and in six nonhuman primate species, using inter-specific consensus intronic primers. The nonhuman primates were of the hominidae, pongidae, hylobatidae, and cercopithecidae families: three chimpanzees (one male and two females; *Pan troglodytes*); one gorilla (*Gorilla gorilla*); one orangutan (*Pongo pygmaeus*), one gibbon (*Hylobates sp*.), one macaque (*Macaca mulatta*) and one baboon (*Papio sp*.). The primer sequences are listed in Additional file 4. Sequencing was performed using standard dye terminator chemistry. Sequences were analysed and polymorphisms detected using the Genalys 3.0 software [42]. Multiple sequence alignments were performed using the ClustalW program [43].

### Modeling analyses

Modeling of the first sushi module of human SRPX2 was undertaken based upon its closest homologue in the Protein Data Bank (PDB) [44,45]: the highest resolved X-ray structure of the first sushi module of complement receptor type 2 (PDB ID: 1LY2) [46], which was identified with a BLAST search [47,48]. The target and template shared 29% sequence identity from N- to C-terminal cysteines. The program Modeller release 8 version 1 [49] was used.

The alignment between the target and template sequences was based on an initial multiple sequence alignment of several individual human sushi-module sequences assigned to cluster-D (with the addition of template sequence CR2 module 1), using the program ProbCons [50] to help position indels more appropriately (see Additional file 2 for target-template alignment). Cluster-D members, according to the recently published convention of sequence assignment [26], are characterized by the presence of a six-cysteine residue motif, forming three putative disulfide bridges. The target-template sequence alignment was subjected to further, minor manual editing guided by positioning of secondary structure elements more appropriately between the target and template sequences; secondary structure was predicted by the server PsiPred version 2.4 [51,52] for the target sequence, or identified by DSSP [53] for the template structure. The additional putative disulfide bridge present in the target (absent in its corresponding template) was restrained during model building. Twenty-five models were generated, and the one with the lowest objective function score [49] selected as the representative model. The loop KGGNY in the hypervariable loop region of SRPX2, for which CR2-template-derived restraints were largely absent was subsequently remodeled under SYBYL version 6.9 (Tripos Associates, St. Louis, MO, USA) after we conducted a loop search for that region against a high resolution subset of the PDB, in order to select the best root-mean square fitting matching loop conformation for that region.

Non-identical side-chain residues of the representative model were optimized using the side-chain replacement program SCWRL version 3 [54]. The model was then protonated and subjected to energy minimization using the Tripos forcefield [55] (20 steps steepest descent followed by 20 steps conjugate gradient) under SYBYL to remove clashes and bad geometries. The model structure was finally checked for valid stereochemistry using PROCHECK version 3.5.4 [56].

### Evolution data analyses

The phylogenetic analysis was performed using the phylogenomic analysis pipeline available in the FIGENIX [57] automated genomic annotation platform [58] with the human SRPX2 protein sequence [Genbank: NP_055282] as input and the NCBI nr or the Ensembl databases for BLAST searches (Additional file 1). The synonymous/non synonymous analyses were conducted in primates assuming the following unrooted tree topology: (Baboon, Macaque, ((((Chimpanzee, Human), Gorilla), Orangutan), Gibbon)). Ancestral sequences were reconstructed with the pamp and codeml programs in the PAML package [59,60], using the parsimony method and the maximum likelihood method, respectively. From these data, the number of synonymous and non-synonymous substitutions and the Ka/Ks ratios were estimated using the DnaSP 4.0 package [61]. The codeml program from the PAML 3.15 packages was used to test for positive selection, using the branch models and the branch-site models, as previously described [62,63] (Additional file 3).

The DnaSP 4.0 program was also used for all population genetic analyses. Nucleotide diversity (π) and Watterson's θ were computed as described [64]. The neutral evolution hypothesis for the SRPX2 intronic polymorphisms was checked with the HKA (Hudson-Kreitman-Aguadé) test [39], using available data on worldwide polymorphisms in non-coding reference (neutral) autosomal and X-linked regions of the human genome (Table 2), as previously described [16].

## Competing interests

The author(s) declares that there are no competing interests.

## Authors' contributions

BR carried out the evolutionary genetics studies in nonhuman primates and in human; performed the statistical analyses; participated in the conception and the design of the study and in the drafting of the manuscript. DCS and PNB carried out the 3-D modeling analysis and participated in the drafting of the manuscript. REB and AB participated in the genetic study of nonhuman primates. PR and ARS participated in the sequence alignments and analyses. AL participated in the statistical analyses. PC participated in the design of the study and in the drafting of the manuscript. PP participated in the design of the study, in the phylogenetic analysis and in the drafting of the manuscript. PS conceived of the study and participated in its design and coordination and in the drafting of the manuscript. All authors read and approved the final manuscript.

## Supplementary Material

Additional file 1 List of the ENSEMBL gene accession numbers. The sequences corresponding to the gene accession numbers were used to construct the phylogenetic tree depicted in Fig. 1. Click here for file

Additional file 2 Target-template alignment used for modelling. This figure represents the output file for optimal sequence alignment between target sushi 1 of SRPX2 and template CR2 sushi module 1. Click here for file

Additional file 3 Analysis of positive selection along the human lineage. The data represent the statistical analysis that was used to test for positive selection, using the branch models and the branch-site models. Click here for file

Additional file 4 List of PCR primers. The '*Exons*' PCR primers were used to amplify exons of the full-length coding region of *SRPX2 *in 12 human females and in six nonhuman primate species. The '*Introns*' PCR primers were used to amplify human genomic fragments from introns 3 and 4 of *SRPX2 *in the same 12 women. Click here for file
