# Supplementary material for: Molecular evolution of the human SRPX2 gene that causes brain disorders of the Rolandic and Sylvian speech areas
Source: BMC Genet. 2007 Oct 18;8:72. doi: 10.1186/1471-2156-8-72 (PMC2151080; doi:10.1186/1471-2156-8-72)
Supplement: Additional file 1 — List of the ENSEMBL gene accession numbers. The sequences corresponding to the gene accession numbers were used to construct the phylogenetic tree depicted in Fig. 1. [file 1471-2156-8-72-S1.doc]

Additional file 1. List of the ENSEMBL gene accession numbers.

1 ENSG00000007908

2 ENSPTRG00000001662

3 ENSGALG00000003399

4 ENSPTRG00000001660

5 ENSG00000174175

6 ENSMMUG00000013044

7 ENSCAFG00000015180

8 ENSBTAG00000020755

9 ENSMUSG00000026580

10 ENSRNOG00000002794

11 NEWSINFRUG00000123102

12 ENSGALG00000002643

13 ENSDARG00000042138

14 NEWSINFRUG00000163702

15 GSTENG00017436001

16 ENSG00000101955

17 ENSCAFG00000013978

18 ENSMMUG00000013135

19 ENSGALG00000016256

20 GSTENG00025984001

21 NEWSINFRUG00000147882

22 ENSDARG00000010318

23 ENSMMUG00000008210

24 ENSG00000102359

25 ENSCAFG00000017527

26 ENSBTAG00000000651

27 ENSMUSG00000031253

28 ENSRNOG00000003715

29 ENSGALG00000006796

30 ENSDARG00000034559

31 GSTENG00031297001

32 ENSRNOG00000033110

33 ENSMUSG00000028369

34 ENSG00000165124

35 ENSPTRG00000021246

36 ENSCAFG00000002928

37 ENSBTAG00000001480

38 NEWSINFRUG00000121360

39 ENSANGG00000004098

40 ENSANGG00000016557
