# Supplementary material for: Molecular evolution of the human SRPX2 gene that causes brain disorders of the Rolandic and Sylvian speech areas
Source: BMC Genet. 2007 Oct 18;8:72. doi: 10.1186/1471-2156-8-72 (PMC2151080; doi:10.1186/1471-2156-8-72)
Supplement: Additional file 2 — Target-template alignment used for modelling. This figure represents the output file for optimal sequence alignment between target sushi 1 of SRPX2 and template CR2 sushi module 1. [file 1471-2156-8-72-S2.doc]

Additional file 2. Target-template alignment used for modelling. BOXSHADE (http://www.ch.embnet.org/software/BOX_form.html) output for optimal sequence alignment between target sushi 1 of SRPX2 and template CR2 sushi module 1. The two domains share 29% sequence identity from N- to C-terminal cysteine residues. The alignment was generated from initial multiple sequence alignments using ProbCons with further manual editing guided by secondary structure. The β-strands of the template structure are indicated by arrows, and additionally, the hypervariable loop labelled to bring out the difference in length for that region between the two sequences. Cysteine residues involved in disulfide bridge formation are connected by lines and labelled above the SRPX2-sushi 1 sequence (the additional disulfide in SRPX2 is shown in red). Identical residues are marked by ‘*’ and conservative substitutions shown by ‘.’ in the consensus line.

Hypervariable loop
