# Supplementary material for: Molecular evolution of the human SRPX2 gene that causes brain disorders of the Rolandic and Sylvian speech areas
Source: BMC Genet. 2007 Oct 18;8:72. doi: 10.1186/1471-2156-8-72 (PMC2151080; doi:10.1186/1471-2156-8-72)
Supplement: Additional file 3 — Analysis of positive selection along the human lineage. The data represent the statistical analysis that was used to test for positive selection, using the branch models and the branch-site models. [file 1471-2156-8-72-S3.doc]

Additional file 3. Analysis of positive selection along the human lineage. The codeml program from the PAML 3.15 packages was used to test the branch and branch-site models. LRT: likelihood ratio test.

Parameters Estimates

| Model | *p* | *l* | Estimates of Parameters | Positively Selected Sites |
| --- | --- | --- | --- | --- |
| Branch models | | | | |
| One-ratio | 1 | -2172.87 | $\hat{w}$ = 0.053 | None |
| Two-ratios | 2 | -2170.44 | $\hat{w}$_0_ = 0.042, $\hat{w}$_1_ = infinity | N/A |
| Two-ratios, ω_1_ =1 | 1 | -2170.84 | $\hat{w}$_0_ = 0.042, $\hat{w}$_1_ = 1.000 | N/A |
| Branch-site models | | | | |
| Model 1a | 1 | -2172.87 | $\hat{p}$_0_ = 1.000 ($\hat{p}$_1_ = 0.000) | Not Allowed |
| Model A | 3 | -2170.44 | $\hat{p}$_0_ = 0.000, $\hat{p}$_1_ = 0.000  ($\hat{p}$_2a_+ $\hat{p}$_2b_ = 1.000) $\hat{w}$_2_ = infinity | 75K (at P>0,09) |
| Model A, ω_2_ =1 | 2 | -2170.84 | $\hat{p}$_0_ = 0.000, $\hat{p}$_1_ = 0.000  ($\hat{p}$_2a_+ $\hat{p}$_2b_ = 1.000) $\hat{w}$_2_ = 1.000 |  |

*p* is the number of free parameters for ω ratios. Parameters in parentheses are presented for clarity only but are not free parameters. Foreground lineage used is human lineage.

where,

One-ratio model assumes the same ω ratio for all branches in the phylogeny;

Two-ratio model assumes that the foreground branches have a ratio ω_1_ that is different from the background ratio ω_0_;

Model1a (NearlyNeutral) assumes two sites classes 0<ω_0_<1 (*p*_0_) and ω_1_=1 (*p*_1_=1- *p*_0_) for all branches;

Model A assumes four sites classes, as described :

| Site Class | Proportion | Background | Foreground |
| --- | --- | --- | --- |
| 0 | *p*_0_ | 0<ω_0_<1 | 0<ω_0_<1 |
| 1 | *p*_1_ | ω_1_=1 | ω_1_=1 |
| 2a | (1- *p*_0-_ *p*_1_) *p*_0_/ (*p*_0_+ *p*_1_) | 0<ω_0_<1 | ω_2_>1 |
| 2b | (1- *p*_0-_ *p*_1_) *p*_0_/ (*p*_0_+ *p*_1_) | ω_1_=1 | ω_2_>1 |

LTR statistics

|  | Null Hypothesis | Alternative Hypothesis | 2ΔlnL | ddl | Pvalue |
| --- | --- | --- | --- | --- | --- |
| Branch models | | | | | |
| Test 1 | One-ratio | Two-ratios | 4,86 | 1 | 0,028* |
| Test 2 | Two-ratios, ω_1_ =1 | Two-ratios | 0,80 | 1 | 0,371 |
| Branch-sites models | | | | | |
| Test 1 | M1a | Model A | 4,84 | 2 | 0,089 |
| Test 2 | Model A, ω_2_ =1 | Model A | 0,80 | 1 | 0,371 |

* Significant (P<5%)
