# Supplementary material for: Molecular evolution of the human SRPX2 gene that causes brain disorders of the Rolandic and Sylvian speech areas
Source: BMC Genet. 2007 Oct 18;8:72. doi: 10.1186/1471-2156-8-72 (PMC2151080; doi:10.1186/1471-2156-8-72)
Supplement: Additional file 4 — List of PCR primers. The 'Exons' PCR primers were used to amplify exons of the full-length coding region of SRPX2 in 12 human females and in six nonhuman primate species. The 'Introns' PCR primers were used to amplify human genomic fragments from introns 3 and 4 of SRPX2 in the same 12 women. [file 1471-2156-8-72-S4.doc]

Additional file 4. List of PCR primers.

|  | **Primer (5’-3’)** |  | **Primer (5’-3’)** |
| --- | --- | --- | --- |
| *Exons* |  | *Intron3:* |  |
| ex2.F | gggaggaaaagggaccataa | Int3A-F | gccctaaatcccagtgacct |
| ex2.R | agtctaactcttccttctccaagtc | Int3A-R | gacctccttccaatcaatcg |
| ex3.F | agttggatgagagggggaag | Int3B-F | aatttttaattttgacaaggtcca |
| ex3.R | aatcacagcccctgattcc | Int3B-R | aaaccccacctcgacctaag |
| ex4 F | tgtcttttcaccaacaccaca | Int3C-F | ttgcagagtcgtatggtaagtg |
| ex4.R | tccgggcatataccctcag | Int3C-R | cccggccagaagaattaaa |
| ex5.F | tctgatgtttctccctgtgct | Int3D-F | ccaaggcgagcagattaaga |
| ex5.R | ggaatgccttctgtggtgag | Int3D-R | gctggtggaaatgcaaaaata |
| ex6/7.F | gtgtgtggcattgcttcagt | Int3E-F | tgacaactgcatagggtcaca |
| ex6/7.R | ggtgggtcagggtagagcag | Int3E-R | caccttgttggctagaatgg |
| ex8.F | gcaccctcttagcctttcct | Int3F-F | ccccttaggatgtgggaact |
| ex8.R | cctgtccatgaggaagatcc | Int3F-R | acatcaaaggggaagcactg |
| ex9.F | atgggagaaacagccagaga | Int3G-F | tctcagcatttagaccgacct |
| ex9.R | tgctgcagagaacaactctga | Int3G-R | aggatgcacacacacagcat |
| ex10.F | cctttcccacactgcttcat | Int3H-F | agcattgtcacatgcctcac |
| ex10.R | aagtttggcagcctccca | Int3H-R | gctgggacctcaacaatcac |
| ex11.F | catgagtggagctgcaaaaa | Int3I-F | gctttggctattctggctct |
| ex11.R | cacccacctcagagtcctgt | Int3I-R | tcctgtttgggtagggtcac |
|  |  | Int3J-F | tcctgaggagttttccctatg |
| *Intron4:* |  | Int3J-R | aaaatgaccatattgctcaaagc |
| Int4A-F | cgattgattggaaggaggtc | Int3K-F | cggagtctcctgacctcgta |
| Int4A-R | atgtttcacagctccactgc | Int3K-R | tgaaattggacacccatctct |
| Int4B-F | tccacttgtggtcaactgct | Int3L-F | caccaccatgcccagcta |
| Int4B-R | gactgcctgctttctggcta | Int3L-R | tcaggggactaataatgtccag |
| Int4C-F | ctggatgcagaagtgctgag | Int3M-F | tcacctgtcttgaccttcca |
| Int4C-R | tgcttggcaaaccagactta | Int3M-R | caggcggatcatttgagg |
| Int4D-F | ggtctttacaaagggacactgg | Int3N-F | cgaatgcagatccctttgat |
| Int4D-R | ggctcatttagggccttagc | Int3N-R | gggaaccatacattgttgatga |
| Int4E-F | gcctggaggagaaaagaagc | Int3O-F | tttgcggtatccatcacctt |
| Int4E-R | tggaattcaggtgacaatgatg | Int3O-R | ggagggaactagcactaccg |
| Int4F-F | atgtacagcctcccctgttg | Int3P-F | gattacaggcgcacactacg |
| Int4F-R | cagcgagagtcaagaagcact | Int3P-R | gactgggaggggtaagtgg |
|  |  | Int3Q-F | tggagtttggagcagacaaa |
|  |  | Int3Q-R | ttgagaccaccctgtcca |
|  |  | Int3R-F | ttccagctcttggactctgg |
|  |  | Int3R-R | taaattgcccaggatcccta |
|  |  | Int3S-F | gccatgcttaattcatctctttc |
|  |  | Int3S-R | acatagcaagtgtgcaatttattt |
|  |  | Int3T-F | tggccaggaaaagtacaacc |
|  |  | Int3T-R | aaatgacatgccaatttgctc |
